# Positive and Negative Psychological Derailment in Chinese Adolescents and the Mechanism by Which It Affects Mental Health: A Mediated Moderation Model

**DOI:** 10.3390/children9050601

**Published:** 2022-04-23

**Authors:** Jinxiong Chu, Ruixiang Gao, Xitong Huang, Lei Mo

**Affiliations:** 1 Key Laboratory of Brain, Cognition and Education Sciences (South China Normal University), Ministry of Education, Guangzhou 510631, China; snsmbnsrcxy@163.com (J.C.); ruixianggao@m.scnu.edu.cn (R.G.); 2019010213@m.scnu.edu.cn (X.H.); 2 School of Psychology, Center for Studies of Psychological Application, and Guangdong Key Laboratory of Mental Health and Cognitive Science, South China Normal University, Guangzhou 510631, China; 3 South Jincai Experimental Junior Middle School, Shanghai 200120, China

**Keywords:** psychological derailment, valence, mental health, mediated moderation model

## Abstract

Psychological derailment refers to the phenomenon whereby original self-expectations are seriously inconsistent with developments in reality. Research to date has neglected the valence of derailment and the mechanism by which it affects mental health. To improve the mental health of Chinese adolescents from the perspective of psychological derailment, after validating the translated Chinese versions of the derailment measurement instruments, we conducted an empirical study on the freshmen in senior high schools and universities in China and obtained three major results. First, the study revealed the prevalence of psychological derailment among Chinese adolescents and its strong correlation with mental health indicators (depression, anxiety, stress and satisfaction with life). Second, the study found significant differences in all mental health indicators among the non-derailed group, the positively derailed group and the negatively derailed group, and suggested that positive psychological derailment may help to ease mental health problems. Third, using path analysis to establish a mediated moderation model relating psychological derailment, psychological derailment valence, self-esteem and depression, the study uncovered that the valence of psychological derailment moderated the effect of psychological derailment on depression, while self-esteem mediated the moderating effect. The implications and limitations of the study are discussed.

## 1. Introduction

On World Mental Health Day 2020 (10 October), China’s National Health Commission released the latest data on mental health in the nation. This shocking report revealed that about 30 million children and adolescents under the age of 17 were suffering from various mood disorders and behavioral problems, including depression, self-injury and Internet addiction [1]. To improve this situation, the Commission issued *A Program to Explore Characteristic Services for the Prevention and Treatment of Depression*. The program proposed to step up interventions for key groups, the first of which was specified to be adolescents. It required all high schools and higher education institutions to include depression screening as a procedure in students’ health examinations [2]. Initiatives such as this program clearly demonstrate the concern of Chinese society for the mental health of young people and students, which is worthy of recognition.

Introduced by Burrow et al. [3], the new concept of “derailment” has sparked great interest in the field of mental health. Derailment often occurs when individuals’ original self-expectations are significantly at odds with their subsequent actual development and has been shown to be a unique indicator of maladjustment. Because adolescents inevitably confront the difference between their expected and actual selves as they grow up, they are at a high risk of psychological derailment. As a derivation of Higgins’ “ideal self” [4], an individual’s expected self is the expectations that they hold for their thoughts and behaviors based on their self-identity. Meanwhile, an individual’s actual self is what they believe to be the actual manifestation of their thoughts and behaviors based on their self-identity. If the difference between the expected self and their actual self is within an acceptable range, the adolescent experiences healthy psychological development. However, if there is a serious inconsistency, the adolescent is psychologically derailed. This psychological derailment leaves them struggling and unable to perceive consistency and continuity in their self, which leads to various forms of maladjustment, such as depression, anxiety and stress. Therefore, it is necessary to explore the phenomenon of psychological derailment and the mechanisms by which it exerts an effect on students’ mental health in the Chinese cultural context. For example, a better understanding of psychological derailment may shed light on how the mental health of Chinese youth can be maintained and improved, how to prevent depression, anxiety and other psychological disorders in this population and reveal new steps for researching psychological derailment.

Therefore, to maintain and improve the mental health of Chinese students, we conducted studies on psychological derailment in this population and cultural context.

## 2. Literature Review

According to Erikson [5], adolescence is a period of “identity crisis”, where “crisis” is not necessarily a time of disaster but rather a necessary turning point or decisive moment of developmental significance. It is the psychological process by which an individual achieves the integration of their self and forms a mature personality. Erikson also pointed out that there are seven main issues to be dealt with during this period: “time perception–time confusion”, “self-certainty–self-doubt”, “role experimentation–role fixation”, “career intentions–career inefficiency”, “gender differentiation–gender confusion”, “power division–power confusion” and “value orientation–value confusion”. If adolescents can develop reasonable responses to these seven issues, their identity crisis is resolved, their identity status is “achieved”, and they can successfully move onto the next stage of psychological development; conversely, if the identity crisis is not resolved, the individual’s status is “diffused” [6], and they cannot proceed in their psychological development. This can have a negative impact on the individual’s well-being and quality of life [7,8]. For example, Huang et al. [9] found that the self-identity crisis scores of college students were significantly and negatively correlated with social adjustment. In its most severe form, a prolonged identity crisis may even signal psychological dysfunction [7].

Although numerous studies have shown that self-identity has an important impact on the psychological health of adolescents, they have focused only on the conceptualization of self-identity and its process as well as statuses [10]. Few scholars have studied the stability of self-identity after its integration, which is an important characteristic of self-identity [11]. However, recent studies have pointed out that in addition to the crises and suffering that may arise during the formation of self-identity, there may be negative consequences when the temporal stability of an established self-identity is disrupted, that is, when individuals feel that their actual development is not in line with the self-expectations that they hold based on their achieved self-identity [3,12,13,14]. To understand the dynamics of self-identity more comprehensively, Burrow et al. [3] developed the concept of “derailment”, which is defined as a state of mind in which an individual recognizes a serious inconsistency between their original self-expectations and subsequent actual development. For example, in a hypothetical scenario, there are two college students who both changed their majors from law to psychology during their sophomore year. While Student A understands the very meaningful connection between who they were as a law student and who they are now as a psychology major, Student B feels that they are no longer moving in the same direction because they find no connection between their past and their present. Although to an outsider these two students experienced the same transition and may be classified as the same type of student, their internal thoughts are very different. Specifically, Student A experiences a greater degree of psychological derailment than Student B.

Using this conceptualization of psychological derailment, Burrow et al. [3] developed the Psychological Derailment Scale and Psychological Derailment Valence Questionnaire. They found that psychological derailment was a better predictor of well-being outcomes than other similar constructs (e.g., self-identity process, self-continuity index, Big Five personality model) [3]. Moreover, in a longitudinal study with college freshmen, they found that the participants’ levels of psychological derailment at college entry significantly predicted their depression level 1.5 years later [3]. This relationship between psychological derailment and depression was later also confirmed by Ratner [15]. Finally, the study [3] also showed that psychological derailment significantly predicted depression, stress and anxiety even when the effect of psychological derailment valence was excluded. That is, even when psychological derailment was positive, such that the individual’s actual subsequent development was better than predicted by their original self-expectations, the above symptoms were still triggered due to the violation of self-consistency [16]. Self-consistency, a concept originally developed by Lecky [17], is based on the central assumption that an individual’s self-concept is essential to survival because it allows them to anticipate and control their social reality to some extent [18,19]. Lecky [17] believed that people strive to obtain information that confirms their existing self-concept to maintain a harmonious and consistent view of themselves.

As suggested by the above studies, studying the concept of psychological derailment and its unique predictive effect on maladaptation may generate valuable insights. Therefore, to maintain and improve the mental health of Chinese adolescent students, it is necessary to study psychological derailment in this population and cultural context. As indicated by Gao et al. [20], the fierce competition among Chinese students within the contemporary Chinese context of exam-oriented education is likely to create great inconsistencies between their original expectations and their actual statuses, thus leading to severe mental maladaptation problems, especially at the transitional grades between the school stages, such as grades 7, 10 and 13 and grades 10 and 13, which, respectively, lie before and after the most fiercely competitive college entrance examination, and thus are particularly noteworthy. To better identify those who are vulnerable to maladjustments such as anxiety and depression so as to take early preventative measures, Chinese versions of the Psychological Derailment Scale and Psychological Derailment Valence Questionnaire should also be developed.

A limitation of previous studies is that they have not paid enough attention to the valence of psychological derailment. Even though Burrow et al. [3] took the valence into account, they partialed this variance out and only focused on the associations between derailment and maladjustment. However, the theory of self-enhancement suggests that psychological derailment valence may have a greater role than has been assumed so far. The theory’s central assumption is that people are motivated to increase their sense of value and therefore seek out information that helps to enhance their view of themselves [18,21]. Therefore, according to this theory, positive psychological derailment is consistent with self-enhancement and leads to positive psychological responses. Furthermore, researchers have shown that individuals respond to external feedback about themselves through two separate channels: cognitive and emotional. Cognitively, they want external feedback to be in harmony with their existing sense of self, while emotionally, they want external feedback to enhance and improve their sense of self. This feedback can affect psychological well-being negatively regardless of whether it is responded to cognitively or emotionally [16,22,23]. Thus, when exploring the effects of psychological derailment on mental health, researchers should not ignore or exclude the valence of psychological derailment but rather explore it in detail.

**Figure 1:**
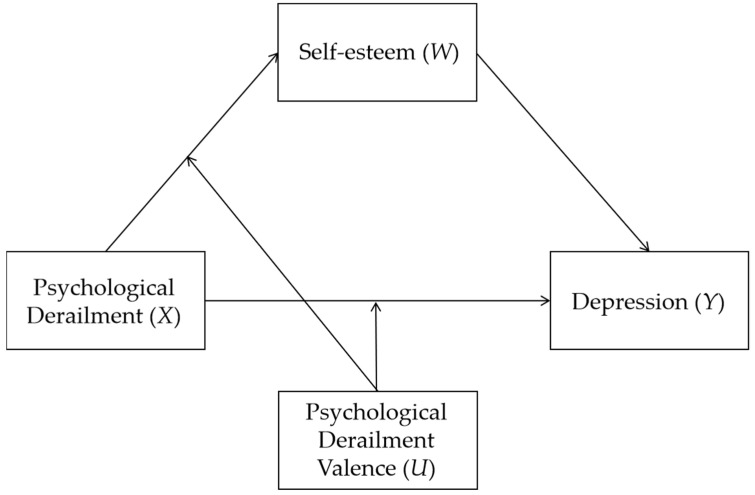
Concept diagram of mediated moderation model relating psychological derailment, valence of psychological derailment, self-esteem and depression.

Finally, the mechanisms underlying the effects of psychological derailment on mental health have not yet been explored. However, doing so is necessary for the prevention of and intervention for psychological derailment. Research on or relating to psychological derailment has focused on its relationships with depression and self-esteem. Shiner pointed out that if an individual has difficulty developing a coherent personal narrative that integrates their sense of self with many of their experiences [24], their self-esteem may be challenged, potentially resulting in feelings of inferiority and depression [25]. Burrow et al. [3] also found a significant negative correlation between psychological derailment and self-esteem. As to the links between self-esteem and depression, a meta-analysis found that compared with other factors of mental health, self-esteem was the factor most strongly correlated with depression, such that the lower the self-esteem, the greater the depression [26]. Furthermore, the vulnerability model of depression states that individuals’ negative self-evaluation, i.e., their low self-esteem, is one of the risk factors leading to depression [27]. Other studies have also shown that self-esteem instability is related to depression and susceptibility to depression [28]. Moreover, self-esteem has been shown to play a mediating role in numerous studies on depression [29,30,31,32,33]. Thus, it can be hypothesized that the valence of psychological derailment moderates the relationship between psychological derailment and depression, and that the moderating effect is mediated by self-esteem (see Figure 1 for the model diagram).

Drawing on the above literature review, we designed an empirical study with three aims to explore (1) the basic circumstances of psychological derailment in Chinese adolescents, (2) the influence of the valence of psychological derailment and (3) the mechanism by which psychological derailment affects mental health. To carry out this research, we first developed reliable and valid survey instruments to measure psychological derailment in Chinese adolescents.

## 3. Methods

### 3.1. Participants

We recruited two samples of Chinese adolescents. The first sample consisted of first-year senior high school students (10th graders) who had just completed junior high school, undergone the Chinese senior high school entrance exams and started their senior high journey to prepare for the college entrance exams. With reference to the previous study [20], we assumed that these recent experiences provided them with a clear frame of reference for assessing the state of their studies and life, and that this frame of reference could help them perceive their psychological derailment. To form this sample, 405 freshmen (aged 14–16) from an ordinary high school in Guangdong province were randomly selected, and 385 students (191 girls) ended up completing the questionnaire, giving a response rate of 95.06%.

The second sample consisted of first-year university students (13th graders) who had just finished high school, undergone the most fiercely competitive college entrance examinations and started a brand-new life stage of higher education dramatically different from elementary education [20]. Similarly, we assumed that they had a clear frame of reference with which to perceive their psychological derailment. To form this sample, 346 freshmen (aged 18–20, considered to be in late adolescence according to [5,34]) from a normal university in Guangdong province were randomly selected, and 310 students (259 females) ended up completing the questionnaire, giving a response rate of 89.60%. The two samples were merged together for analyses.

### 3.2. Procedures and Variables

The questionnaire was administered for the first sample by their mental health teachers on paper. Meanwhile, the second sample of participants completed the questionnaire themselves online using Wenjuanxing (https://www.wjx.cn/, accessed on 6 May 2020), which recompensed them with random rewards. The questionnaire consists of eight scales: (1) the Chinese version of Psychological Derailment Scale (CPDC), (2) the Chinese version of Psychological Derailment Valence Questionnaire (CPDVQ), (3) Ego Identity Process Questionnaire (EIPQ), (4) Positive Effect and Negative Effect Scale (PANAS), (5) Depression, Anxiety and Stress Scale with 21 items (DASS-21), (6) Satisfaction with Life Scale (SLS), (7) Center for Epidemiologic Studies Depression Scale (CES-D) and (8) Rosenberg Self-Esteem Scale (RSE). The third and fourth scales were used as effective standards to validate the two self-translated Chinese versions of psychological derailment survey instruments only and excluded from the formal analyses. It took the participants around 30 min to complete the eight scales.

#### 3.2.1. Psychological Derailment

We invited three experts on the development of the CPDC. The first expert, who had a master’s degree in psychology and a TEM-8 certificate, translated the original version of Psychological Derailment Scale [3]; then, the second expert, who had a master’s degree in psychology and a band-7 in IELTS, modified the translated version; finally, the third expert, who had a master’s degree in translation, proofread it. The CPDC consists of 10 items (Items 1, 2, 5 and 10 scored in reverse), such as “I see myself now as the person I always thought I would be”. The items were scored on a 5-point Likert scale ranging from 1, “strongly disagree” to 5, “strongly agree”. The reliability and validity of the CPDC are reported in the results section.

#### 3.2.2. Psychological Derailment Valence

We went through a similar process for the development of the CPDVQ, which required the participants to “think about who you are now based on a comparison with who you were in the past” in the following two aspects: sense of self-concept and sense of direction in life, each of which has only one item. The participants rated the extent to which they had changed on a scale ranging from −3, “extremely negative change” to 3, “extremely positive change”, where 0 represented no detectable change. The reliability and validity of the CPDVQ are reported in the results section as well.

#### 3.2.3. Self-Consistency

Referring to the previous study [3], we adapted the EIPQ [35] to validate the content of the CPDC. The EIPQ consists of two subscales of exploration and commitment and 37 items in total, 16 for the former subscale and 21 for the latter. Aside from four items that were scored in reverse, the other items were scored on a 6-point scale ranging from 1, “strongly disagree” to 6, “strongly agree”. In this study, the Cronbach’s α for the subscale of exploration, the subscale of commitment and the whole scale were 0.817, 0.808 and 0.843, respectively.

#### 3.2.4. Positive and Negative Effect

Referring to previous studies [3,16], we adapted the PANAS [36] to validate the content of the CPDVQ. On the PANAS, the participants were asked to rate the frequency of 10 positive emotions (e.g., “interested”) and 10 negative emotions (e.g., “upset”) they had recently experienced on a 5-level scale ranging from 1, “very slightly or not at all” to 5, “extremely”. In this study, the Cronbach’s α for the subscale of positive effect was 0.845 and 0.886 for the subscale of negative effect.

#### 3.2.5. Depression, Anxiety and Stress

On the DASS-21 [37], the participants rated the extent to which each of the items had applied to them in the past week, using a scale that ranged from 0, “does not fit” to 3, “always fits”. The subscale for stress includes seven items, such as “I find it difficult to calm myself down” and “I tend to react to things with excessive sensitivity” (α = 0.787, the current study); the subscale for anxiety has seven items, such as “I feel thirsty” and “I am worried about situations where I might panic or make a fool of myself” (α = 0.800, the current study); and the subscale for depression has seven items, such as “I feel that I have nothing to look forward to in the near future” and “I cannot feel enthusiastic about anything” (α = 0.839, the current study).

#### 3.2.6. Satisfaction with Life

On the SLS [38] that contains five items, the participants rated items such as “In most ways my life is close to my ideal” on a 7-point Likert scale ranging from 1, “strongly disagree” to 7, “strongly agree” (α = 0.794, the current study).

#### 3.2.7. Depression

For the accurate measurement of depression, we used the CES-D [39], for which the participants rated 20 items with respect to how often each was felt or experienced on a scale ranging from 1, “less than 1 day” to 4, “5–7 days”. Items 4, 8, 12 and 16 were scored in reverse. The total score ranged from 0 to 60, with a higher score indicating a higher level of depression. Sample items include “I have been bothered by things that didn’t bother me before” and “I feel depressed, even with the help of family and friends” (α = 0.915, the current study).

#### 3.2.8. Self-Esteem

For the measurement of self-esteem, we used the RSE [40], which consists of 10 items such as “Overall, I feel very satisfied with myself”. The participants rated the items on a 4-point Likert scale ranging from 1, “strongly disagree” to 4, “strongly agree”. Items 1, 2, 4, 6 and 7 were scored in the forward direction, while the rest were scored in reverse. The total score ranged from 10 to 40 (α = 0.885, the current study).

## 4. Data Analyses and Results

### 4.1. Validation of the CPDC and CPDVQ

For reliability, in this study, the CPDC had an acceptable Cronbach’s α of 0.671 and the CPDVQ had a satisfactory Cronbach’s α of 0.795, which reflected the two translated Chinese scales had a fair internal consistency.

For construct validity, confirmatory factor analysis (CFA) was conducted to load all 10 items of the CPDC into one factor, and the result shows that: *χ*^2^/*df* = 13,524.109/5414 = 2.498 < 3, RMSEA = 0.046 < 0.05, CFI = 0.745 > 0.7, indicating an acceptable construct validity. The CPDVQ had only one item for each of the two subscales and was thus unsuitable for CFA. For content validity, the scores on the CPDC positively correlated with the scores on the subscale of exploration in the EIPQ and negatively correlated with commitment (see Table 1), which was consistent with a previous study [3]; the scores on the CPDVQ negatively correlated with the scores of the CPDC and the subscale of negative effect in PANAS (see Table 2), and positively correlated with positive effect, which was consistent with previous studies [3,16] as well.

Therefore, the CPDC and CPDVQ were reliable and valid. For the final scores of the CPDC and CPDVQ, we, respectively, calculated the mean of the scores of the ten items in the CPDC and the mean of the scores of the two single items in the CPDVQ.

### 4.2. The Basic Circumstances of Psychological Derailment in Chinese Adolescents

We divided the participants into two groups according to their scores on the CPDC. Specifically, they were assigned to the non-derailed group if the interval of their scores was [1,3], and assigned to the derailed group if their score interval was [3,5]. The results show that 49.8% of the participants reported psychological derailment, which reflected the prevalence of psychological derailment among Chinese students and alerted our attention. The results of our chi-squared test show that there was no significant difference in gender (*χ*^2^ = 0.000, *p* = 0.996) or grade level (*χ*^2^ = 0.166, *p* = 0.684) between the non-derailed group and the derailed group, which was consistent with the previous result [3].

Then, we conducted T-tests to evaluate the impact of psychological derailment on Chinese adolescents’ mental health and psychological well-being (see Table 3). The difference in depression between the non-derailed group and the derailed group was significant, with the degree of depression in the non-derailed group being significantly lower than that in the derailed group. The difference in anxiety was also significant, with the degree of anxiety of the non-derailed group being significantly lower than that of the derailed group. The difference in stress was likewise significant, with the degree of stress of the non-derailed group being significantly lower than that of the derailed group. Last, the life satisfaction of the non-derailed group was significantly higher than that of the derailed group. These results are consistent with the previous finding [3] that psychological derailment is harmful to adolescents’ psychological health and well-being.

### 4.3. The Influence of the Valence of Psychological Derailment

We divided the participants into three groups according to their scores on the CPDC and CPDVQ altogether. Those with psychological derailment scores ≤ 3 or psychological derailment valence scores = 0 (*N* = 385, accounting for 55.3% of the total participants) were classified as the non-derailed group; those with positive psychological derailment valence scores were classified as the positively derailed group (*N* = 212, accounting for 30.5% of the total participants), while the participants with negative valence scores were classified as the negatively derailed group (*N* = 98, or 14.1% of the total participants).

Then, we conducted F tests to see the effect of the valence of psychological derailment on Chinese adolescents’ mental health and psychological well-being (see Table 4). There were significant differences in depression among the three groups: *F*(2, 692) = 94.555, *p* < 0.001, *η*^2^ = 0.21. The post hoc test revealed that the depression level of the negatively derailed group was significantly higher than that of the non-derailed group and the positively derailed group (*p* < 0.05), while there was no significant difference between the non-derailed group and the positively derailed group (*p* = 0.216).

Similarly, there were significant differences in anxiety among the three groups: *F*(2, 692) = 42.090, *p* < 0.001, *η*^2^ = 0.11. The post hoc test revealed that the anxiety level of the negatively derailed group was significantly higher than that of the non-derailed group and positively derailed group (*p* < 0.001), while the anxiety level of the positively derailed group was marginally significantly lower than that of the non-derailed group (*p* = 0.068).

Likewise, the differences in stress were significant among the three groups: *F*(2, 692) = 47.984, *p* < 0.001, *η*^2^ = 0.12. The post hoc test revealed that the stress level of the negatively derailed group was significantly higher than that of the non-derailed group and positively derailed group (*p* < 0.001), while the stress level of the positively derailed group was significantly lower than that of the non-derailed group (*p* = 0.007).

Last, there were also significant differences in life satisfaction among the three groups: *F*(2, 692) = 37.364, *p* < 0.001, *η*^2^ = 0.10. The post hoc test revealed that the life satisfaction level of the negatively derailed group was significantly lower than that of the non-derailed group and positively derailed group (*p* < 0.001), while the life satisfaction level of the positively derailed group was marginally significantly higher than that of the non-derailed group (*p* = 0.092).

These results are basically consistent with the self-enhancement theory, which states that positive external feedback helps individuals maintain positive emotional responses (rather than cognitive responses) [16,18,21,22,23]. Moreover, the mental state of the positively derailed group was healthier than that of the non-derailed group, which inspired us to pay attention to the gratifying role of positive psychological derailment in relieving mental health problems.

### 4.4. The Mechanism by Which Psychological Derailment Affects Mental Health

As we have illustrated our hypothesis in the literature review section, we used depression measured by CES-D as the major indicator of mental health to investigate how psychological derailment and its valence together influence students’ mental health, that is, to test the mediated moderation model (see Figure 1).

Table 5 displays the Pearson correlation coefficients among the four variables. Psychological derailment, psychological derailment valence and self-esteem were significantly correlated with depression (*p* < 0.01). There was a significant but weak negative correlation between psychological derailment and psychological derailment valence (*p* < 0.01). Both psychological derailment and psychological derailment valence were significantly correlated with self-esteem (*p* < 0.01). According to Wen et al. [41], these results show that the variables were suitable for the following path analysis.

**Figure 2:**
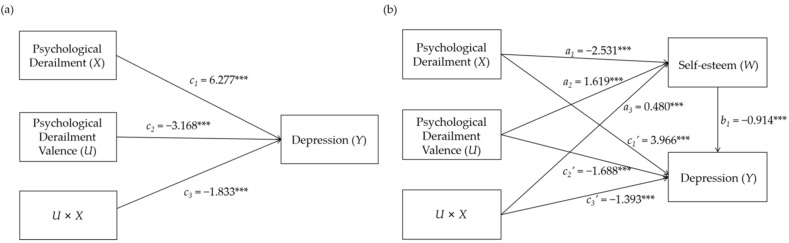
Results of the path analysis to test the moderating effect of psychological derailment valence (**a**) and the mediating effect of self-esteem (**b**). *** *p* < 0.001.

We used Mplus 8.3 to conduct the path analysis. As suggested by Ye and Wen [42], the first step was to analyze the moderating effect of psychological derailment valence (*U*) on the relation between depression (*Y*) and psychological derailment (*X*) by regressing *Y* on *X*, *U* and the interaction term (*UX*); the significant coefficient (*c_3_* = −1.833, *t* = −4.522, *p* < 0.001) associated with *UX* implies that the moderating effect was significant (see Figure 2a). In order to further uncover the essence of this moderating effect, we conducted a simple slope test with a reference to Fang et al. [43]; the result shows that when the valence of psychological derailment was positive, the participants with high levels of psychological derailment showed only a slight increase in depression (*b* = 0.207, *t* = 4.676, *p* < 0.001) compared with those with low levels of psychological derailment and that when the valence of psychological derailment was negative, the participants with high levels of psychological derailment showed an obvious increase in depression (*b* = 0.462, *t* = 11.967, *p* < 0.001) compared with those with low levels of psychological derailment; that is, positive psychological derailment attenuated the risk of depression from psychological derailment, which confirmed the result in Section 4.3.

Then, we continued to test the mediating effect of self-esteem (*W*) by regressing *W* on *X*, *U* and *UX* and regressing *Y* on *X*, *U*, *W*, *UX* and *UW*; the coefficient (*a*_3_ = 0.480, *t* = 2.133, *p* < 0.05) from *UX* to *W* and the coefficient (*b*_1_ = −0.914, *t* = −15.515, *p* < 0.001) from *W* to *Y* were both significant, which implied that the moderated effect of *U* on the relation between *Y* and *X* was mediated by *W*. Additionally, the moderated effect was partially mediated because the coefficient (*c*_3_′ = −1.393, *t* = −3.976, *p* < 0.001) from *UX* to *Y* was still significant (see Figure 2b). In addition, the result of the simple slope test demonstrates that when the valence of psychological derailment was positive, the participants with high levels of psychological derailment showed only a slight decrease in self-esteem (*b* = −0.200, *t* = −4.169, *p* < 0.001) compared with those with low levels of psychological derailment and that when the valence of psychological derailment was negative, the participants with high levels of psychological derailment showed an obvious decrease in self-esteem (*b* = −0.331, *t* = −7.900, *p* < 0.001) compared with those with low levels of psychological derailment; that is, positive psychological derailment reduced the risk of a decline in self-esteem from psychological derailment, which again confirmed the result in Section 4.3.

## 5. Discussion

Psychological derailment is a phenomenon that often occurs in the development of adolescents when there is a serious inconsistency between their original self-expectations and developments in reality. Research has considered its negative effect on students’ mental health but not its valence and mechanisms. With the aim of improving the mental health of Chinese adolescents from the perspective of psychological derailment, this current study conducted in China, which recruited the freshmen in senior high schools and universities as participants, obtained three major results.

First, the study revealed that, in general, psychological derailment was prevalent among the Chinese adolescent students. This may be explained by various reasons. In terms of environmental factors, it may be related to the lack of communication between administrators of different stages of education and the independent curricula as well as the students’ adjustment to their new environments and new people [20,44,45]. This finding suggests that researchers should pay more attention to students’ expectations and self-identity when considering the development of their mental health.

Second, the study found that there were significant differences in all mental health indicators among the non-derailed group, the positively derailed group and the negatively derailed group. This finding, to some extent, makes a contribution to the literature on psychological derailment by refuting the findings of Burrow et al. [3], who concluded that valence had no influence on the effects of psychological derailment. Furthermore, the current study found that the positively derailed group had the lowest stress levels of the three groups, which may be because they experienced the most satisfaction with themselves, suggesting that psychological derailment was not without merits at all.

Third, the study further explored the mechanism underlying the relationship between psychological derailment and depression. The results of the mediation analysis show that psychological derailment had a direct effect on depression, as well as an indirect effect on depression through self-esteem. This may be because changes in self-esteem levels are triggered by changes in adolescents’ thinking and integration of self over time [46,47]. Meanwhile, the results of the moderation analysis indicate that psychological derailment valence had a risk-mitigating moderating effect on the abovementioned mediation relationship. This means that although psychological derailment has a critical influence on depression, the adolescent still adjusts well socially and is less likely to experience depression if the perceived valence of psychological derailment is positive, that is, if the adolescent perceives their life direction to be positive and their self-concept to be improved. This, again confirming the second result, suggests that for both self-reflection and mental health education, researchers should inspect not only the magnitude of individual self-inconsistency (psychological derailment) but also the direction of this inconsistency (psychological derailment valence). They should pay more attention to how positive psychological derailment may help adolescents achieve emotional stability and social adjustment. This finding on psychological derailment valence also strongly supports the theoretical stance of considering self-enhancement with self-concordance to understand the complex responses of individuals to external feedback.

Overall, our findings have the following important implications for the mental health care of Chinese adolescents in education. First, the CPDS and CPDVQ should be included in school psychological surveys. The existing surveys generally use tools such as Symptom Checklist-90 (SCL-90), Self-Rating Depression Scale (SDS) and CES-D, which designate obvious symptoms as indicators and cannot identify mental health problems such as early depressive symptoms. Incorporating the CPDS and CPDVQ into school psychological surveys would help educators detect, prevent and intervene in students’ mental health problems in the early stages. Second and simultaneously, school teachers should improve their sensitivity to students’ psychological derailment, while relative counseling should be integrated into school mental health care systems. Finally, the meaning of positive psychological derailment and the mediating effect of self-esteem should be communicated to teachers. When negative psychological derailment occurs, the teacher’s care for the student’s self-esteem is essential.

However, there are some limitations to our study. We conducted a cross-sectional study, meaning that it could not provide insight into the dynamic impact of psychological derailment on the depression of adolescent students. Future researchers could therefore track the conditions of participants over time. Furthermore, the self-report method used in our data collection may have introduced a certain degree of social desirability bias into our data. Therefore, researchers could combine different data collection methods, e.g., the Word Association Test and Implicit Association Test [20], to balance out such bias in future studies. Last, although we investigated the mediating role of self-esteem and the moderating role of psychological derailment valence in the relationship between psychological derailment and depression, more research is needed to shed light on whether there are other variables involved in this mechanism.

## Figures and Tables

**Table 1:** The correlation between CPDC and EIPQ.

	EIPQ	
	Exploration	Commitment
CPDC	0.214 **	−0.283 **

** *p* < 0.01.

**Table 2:** The correlation among CPDVQ, CPDC and PANAS.

		PANAS	
	CPDC	Negative Effect	Positive Effect
CPDVQ	−0.230 **	−0.356 **	0.456 **

** *p* < 0.01.

**Table 3:** The impact of psychological derailment on Chinese adolescents’ mental health and psychological well-being.

Mental Health Indicators	Group	*M*	*SD*	*t*	*p*	Cohen’s *d*
Depression	derailed	4.01	3.954	−7.583	0.000 ***	0.78
	non-derailed	2.13	2.414			
Anxiety	derailed	5.65	3.989	−6.783	0.000 ***	0.62
	non-derailed	3.86	2.900			
Stress	derailed	6.03	4.087	−7.327	0.000 ***	0.66
	non-derailed	4.04	3.000			
Life satisfaction	derailed	18.31	5.661	6.474	0.000 ***	0.49
	non-derailed	21.09	5.635			

*** *p* < 0.001.

**Table 4:** The effect of the valence of psychological derailment on Chinese adolescents’ mental health and psychological well-being.

Mental Health Indicators	Group	*M*	*SD*	*F*	*p*	*η* ^2^
Depression	Negatively derailed	6.94	4.499	94.555	0.000 ***	0.21
	Positively derailed	2.64	2.910			
	Non-derailed	2.32	2.583			
Anxiety	Negatively derailed	7.61	4.503	42.090	0.000 ***	0.11
	Positively derailed	4.09	3.049			
	Non-derailed	4.62	3.419			
Stress	Negatively derailed	8.10	4.385	47.984	0.000 ***	0.12
	Positively derailed	4.24	3.157			
	Non-derailed	5.05	3.594			
Life satisfaction	Negatively derailed	15.32	5.216	37.364	0.000 ***	0.10
	Positively derailed	20.71	5.671			
	Non-derailed	19.91	5.405			

*** *p* < 0.001.

**Table 5:** Descriptive statistics and correlation analyses of psychological derailment, psychological derailment valence, self-esteem and depression.

	*M*	*SD*	Psychological Derailment	Depression	Self-Esteem	Psychological Derailment Valence
Psychological Derailment	3.036	0.5543	1			
Depression	15.16	10.180	0.454 **	1		
Self-Esteem	28.65	5.202	−0.370 **	−0.674 **	1	
Psychological Derailment Valence	0.845	1.2166	−0.230 **	−0.521 **	0.473 **	1

** *p* < 0.01.

## Data Availability

The data that support the findings of the study are available on request from the corresponding author. The data are not publicly available due to privacy or ethical restrictions.
